# Patient outcome after pulmonary radiofrequency ablation: a prospective analysis of results from a single centre

**DOI:** 10.1186/1470-7330-14-S1-P8

**Published:** 2014-10-09

**Authors:** C Zagorski, S Forbes, PE Jennings, R Soomal, SL Smith

**Affiliations:** 1 Ipswich Hospital, Ipswich, UK

## Aim

To assess the safety of pulmonary RFA and analyse the factors which affect overall survival and disease-free survival in patients with pulmonary malignancy treated with ablation.

## Materials and methods

90 tumours in 45 patients who were treated between April 2009 and August 2013 during 83 separate procedures were analysed using a prospective database. Patient records and imaging was reviewed on the hospital PACS system. For each patient we recorded the histologically confirmed nature of the primary tumour, site and number of metastases and if the patient received prior chemotherapy or other treatments. On a lesion by lesion basis we recorded the site and size of the lesion, ablation details, lesional progression-free survival, complications and overall survival. Factors were compared by the log rank test and Kaplan-Meier survival analysis was performed.

## Results

The median overall survival was 105 weeks from initial RFA, with 1, 2 and 3 years survival of 95.5%, 89.9% and 77.7% respectively. 15 lesions (17%) developed recurrence; with the mean time to recurrence being 32 weeks. 40% of those with recurrence occurred when the lesion was abutting a major vessel. Repeat RFA in 8 of these 15 lesions demonstrated no further recurrence in 7. The rate of significant complications (those requiring treatment), was 12%. Patients with 3 or more lesions ablated were found to have a mean survival of 150 weeks, compared to 195 weeks in those with a total number of lesions ≤ 2. This came close to statistical significance (p=0.091).

## Conclusion

Three-year survival of 77.7% compares favourably with surgery. Patients with 3 or more lesions treated are likely to have reduced survival than those with 2 or fewer lesions treated.

